# Differences in lipid metabolism between anagliptin and sitagliptin in patients with type 2 diabetes on statin therapy: a secondary analysis of the REASON trial

**DOI:** 10.1186/s12933-019-0965-3

**Published:** 2019-11-16

**Authors:** Atsuko Chihara, Atsushi Tanaka, Takeshi Morimoto, Mio Sakuma, Michio Shimabukuro, Takashi Nomiyama, Osamu Arasaki, Shinichiro Ueda, Koichi Node

**Affiliations:** 1 Department of Cardiovascular Medicine, Saga University, 5-1-1 Nabeshima, Saga, 849-8501 Japan; 2 Department of Clinical Epidemiology, Hyogo College of Medicine, Nishinomiya, Japan; 3 Department of Diabetes, Endocrinology and Metabolism, Fukushima Medical University, Fukushima, Japan; 4 Department of Endocrinology and Diabetes Mellitus, Fukuoka University, Fukuoka, Japan; 5 Department of Cardiology, Tomishiro Central Hospital, Tomigusuku, Japan; 6 Department of Pharmacology and Therapeutics, University of the Ryukyus, Nishihara, Japan

**Keywords:** Type 2 diabetes, Anagliptin, Sitagliptin, Lipid metabolisms, Lathosterol

## Abstract

**Background:**

Anagliptin, a dipeptidyl peptidase-4 inhibitor, is reported to reduce the level of low-density lipoprotein cholesterol (LDL-C). The underlying mechanism of this effect and effect on lipid metabolism however remains uncertain.

**Aim and methods:**

We therefore evaluate the effects of anagliptin on lipid metabolism-related markers compared with those of sitagliptin. The study was a secondary analysis using data obtained from the Randomized Evaluation of Anagliptin versus Sitagliptin On low-density lipoproteiN cholesterol in diabetes (REASON) trial. This trial in patients with type 2 diabetes at a high risk of cardiovascular events and on statin therapy showed that anagliptin reduced LDL-C levels to a greater extent than sitagliptin. Cholesterol absorption (campesterol and sitosterol) and synthesis (lathosterol) markers were measured at baseline and 52 weeks in the study cohort (n = 353).

**Results:**

There was no significant difference in the changes of campesterol or sitosterol between the two treatment groups (*p* = 0.85 and 0.55, respectively). Lathosterol concentration was increased significantly at 52 weeks with sitagliptin treatment (baseline, 1.2 ± 0.7 μg/mL vs. 52 weeks, 1.4 ± 1.0 μg/mL, *p* = 0.02), whereas it did not change in the anagliptin group (baseline, 1.3 ± 0.8 μg/mL vs. 52 weeks, 1.3 ± 0.7 μg/mL, *p* = 0.99). The difference in absolute change between the two groups showed a borderline significance (*p *= 0.06).

**Conclusion:**

These findings suggest that anagliptin reduces LDL-C level by suppressing excess cholesterol synthesis, even in combination with statin therapy.

*Trial registration* ClinicalTrials.gov number NCT02330406. https://clinicaltrials.gov/ct2/show/NCT02330406; registered January 5, 2015.

## Background

Strict management of lipid profiles is a clinically critical issue to prevent development and recurrence of cardiovascular events especially in high risk patients, such as those with diabetes [1]. The Randomized Evaluation of Anagliptin versus Sitagliptin On low-density lipoproteiN cholesterol in diabetes (REASON) trial in patients with type 2 diabetes (T2D) at high risk of cardiovascular events and a serum low-density lipoprotein cholesterol (LDL-C) level > 100 mg/dL under statin treatment demonstrated that 52 weeks of anagliptin treatment was associated with a greater reduction in serum LDL-C levels compared to those observed with sitagliptin [2]. The estimated treatment difference between the two groups was − 4.52 mg/dL (95% confidential interval − 8.02 to − 1.02). This finding suggests that anagliptin treatment is clinically useful for further reducing LDL-C in T2D patients who require aggressive LDL-C-lowering treatment.

Although several previous studies have also reported similar effects for anagliptin [3, 4], the underlying mechanisms of its LDL-C-lowering effect and influence on lipid and other metabolisms have yet to be fully understood. Furthermore, little is known regarding differences from other types of dipeptidyl peptidase-4 (DPP-4) inhibitors. To better understand the clinical effects of anagliptin, we performed a secondary analysis using data obtained from the REASON trial.

It is well known that statin therapy can alter markers of LDL-C lowering [5, 6]. Regarding the effect of anagliptin on those markers, only the single-arm pilot study of Aoki et al. [7] reported that 4 weeks of anagliptin therapy decreased the cholesterol synthesis marker, lathosterol, without changing cholesterol absorption markers. In the current analysis we also attempted to investigate the effects of anagliptin, compared to sitagliptin, on these markers to explore the possible mechanisms of anagliptin-mediated LDL-C lowering seen in the REASON trial. This trial also examined differences in the effects of anagliptin and sitagliptin on several metabolic markers, including those involved in lipid metabolism.

## Methods

### Trial design and patients

The detailed design of the REASON trial has been published elsewhere [8]. Briefly, the trial was a multicenter, randomized, open-label, parallel-group design that assessed the effect of anagliptin (100 mg, twice daily), relative to sitagliptin (50 mg once daily), on reduction in LDL-C in adult patients with T2D at high risk of cardiovascular events and whose LDL-C levels were > 100 mg/dL despite treatment with a statin. Eligible patients had any one of previously documented atherosclerotic lesions in the coronary, intracranial, carotid, or other peripheral arteries. Randomization was performed centrally through a web-based system using a stochastic minimization algorithm balanced for hospitals, HbA1c (≥ 8.0%, < 8.0%), use of DPP-4 inhibitors prior to randomization, sex, body mass index (≥ 25 kg/m^2^, < 25 kg/m^2^), and LDL-C (≥ 130 mg/dL, < 130 mg/dL). The trial was conducted in accordance with the Declaration of Helsinki and the Ethical Guidelines for Medical and Health Research Involving Human Subjects in Japan. The institutional review boards at the University of the Ryukyus and each participating center approved the trial. All enrolled patients provided written informed consent prior to randomization. The trial was registered on Clinicaltrials.gov (NCT02330406).

### Measurements

The clinical characteristics of the study cohort (n = 353; anagliptin 177, sitagliptin 176) were evaluated at baseline. The following laboratory parameters were also obtained at baseline and 52 weeks, with the analyses carried out at a core laboratory (SRL Inc., Tokyo, Japan); LDL-C measured by the direct method, total cholesterol, high-density lipoprotein cholesterol, triglyceride, campesterol, sitosterol, lathosterol, high-molecular weight adiponectin, and FIB-4 index calculated as age × aspartate aminotransferase (U/L)/[platelet (10^9^/L) × alanine aminotransferase^1/2^ (U/L)] [9]. In addition, the stored serum samples obtained from a randomly selected cohort in the REASON trial (n = 100; anagliptin 50, sitagliptin 50) were used to measure remnant-like particle cholesterol (RLP-C), malondialdehyde-modified low-density lipoprotein (MDA-LDL), and lipoprotein (a). The analyses were carried out at a core laboratory (LSI Medience Corp., Tokyo, Japan).

### Statistical analysis

The analyses were performed on the full analysis set, which included participants who received an allocated treatment, provided assessable outcome data, and were managed under the intention-to-treat principle. Categorical variables were expressed as frequencies with percentages, and continuous variables as means with standard deviation. We compared the changes from baseline to 52 weeks in the groups using the paired t-test and differences in changes between the two treatment groups using the two-sample t-test. All statistical analyses were performed at the data center (Institute for Clinical Effectiveness) by the study statisticians (Morimoto T and Sakuma M), using JMP 13.1 (SAS Institute Inc, Cary, NC) and SAS 9.4 (SAS Institute Inc, Cary, NC). All P values were two-sided, with P < 0.05 considered to be statistically significant.

## Results

### Baseline characteristics of patients

The baseline clinical characteristics were comparable in the two treatment groups in both the study and randomly selected cohorts (Table 1). In the study cohort (n = 353 patients), the mean age was 68 years, 76% had hypertension, and 45% had a previous history of coronary artery disease. Of these patients, 79% had received a strong statin and 8% had received ezetimibe.

**Table 1:** Baseline demographic and clinical characteristics

	Overall cohort (*N* = 353)		Randomly extracted cohort (*N* = 100)	
	Anagliptin (*n* = 177)	Sitagliptin (*n* = 176)	Anagliptin (*n* = 50)	Sitagliptin (*n* = 50)
Age, year	68 ± 10	68 ± 9	67 ± 8	68 ± 9
Female	67 (38)	72 (41)	19 (38)	18 (36)
Body mass index, kg/m^2^	26.5 ± 4.0	25.9 ± 3.5	26.1 ± 3.5	24.7 ± 2.7
Systolic blood pressure, mm Hg	134 ± 16	132 ± 16	130 ± 14	134 ± 19
Diastolic blood pressure, mm Hg	74 ± 12	71 ± 11	70 ± 11	71 ± 10
Smoker	92 (52)	103 (59)	26 (52)	31 (62)
Current smoker	30 (17)	24 (14)	7 (14)	8 (16)
Past smoker	62 (35)	79 (45)	19 (38)	23 (46)
Non-drinker	102 (58)	102 (58)	35 (70)	26 (52)
Hypertension	137 (77)	133 (76)	38 (76)	36 (72)
Coronary artery disease	80 (45)	79 (45)	23 (46)	19 (38)
Stroke	26 (15)	26 (15)	7 (14)	5 (10)
Treatment				
Renin–angiotensin–aldosterone system inhibitor	107 (60)	99 (56)	29 (58)	27 (54)
Strong statin^a^	142 (80)	136 (77)	37 (74)	39 (78)
Ezetimibe	18 (10)	12 (7)	6 (12)	1 (2)
Metformin	87 (49)	84 (48)	26 (52)	18 (36)
Sulfonylurea	46 (26)	37 (21)	13 (26)	10 (20)
Thiazolidine	25 (14)	31 (18)	9 (18)	10 (20)
Insulin	13 (7)	15 (9)	4 (8)	7 (14)
Other glucose-lowering drugs	27 (15)	18 (10)	9 (18)	5 (10)

Data are expressed as mean ± standard deviation or n (%)

^a^Indicates atorvastatin, rosuvastatin, and pitavastatin

### Changes in markers of cholesterol absorption and synthesis

**Fig. 1:**
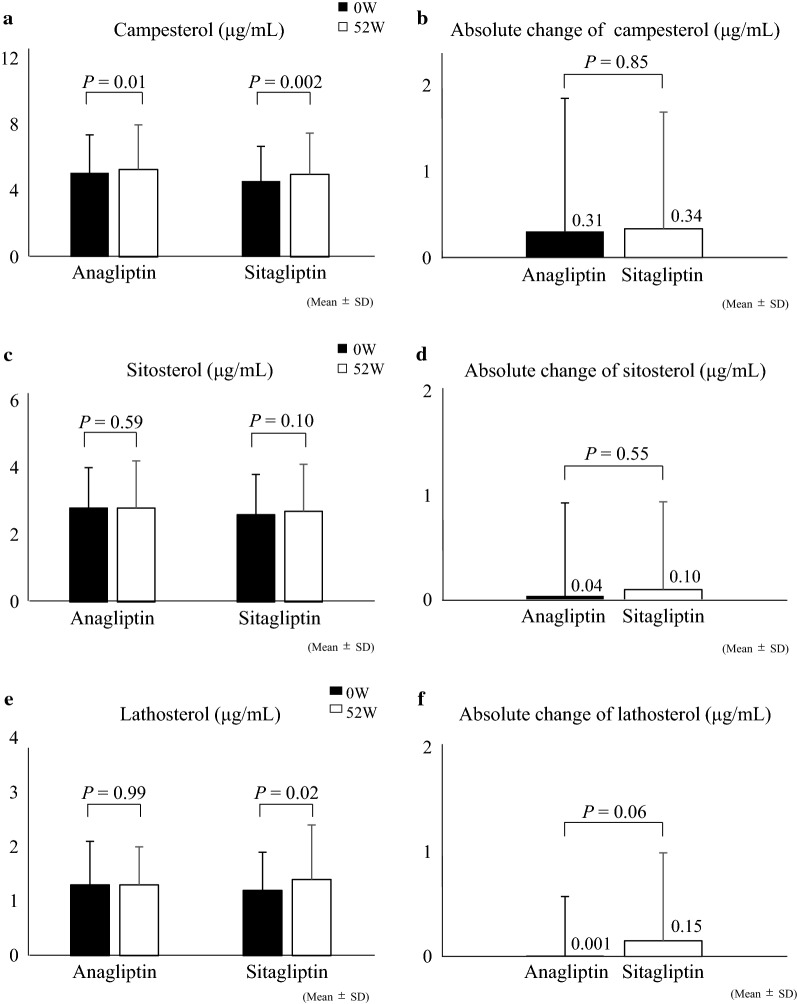
Changes in the markers of lipid metabolism. Comparison of **a** campesterol, **c** sitosterol, **e** lathosterol at 0 and 52 weeks and absolute change in **b** campesterol, **d** sitosterol, **f** lathosterol at 52 weeks

Figure 1 shows the changes in the markers of cholesterol absorption (campesterol and sitosterol) and synthesis (lathosterol) from baseline to 52 weeks. Campesterol levels increased in both treatment groups, with no significant difference in the absolute changes between the two groups (Fig. 1a, b). Similarly, there was no significant change in sitosterol levels and no group different in the absolute changes (Fig. 1c, d). On the other hand, lathosterol increased significantly in the sitagliptin groups, while there was no change in the anagliptin groups (Fig. 1e). The difference in absolute change between the groups showed borderline significant (Fig. 1f). In patients who had not received ezetimibe therapy at baseline (Additional file 1), the trend of changes in these markers was also similar to those observed in the original overall cohort (Fig. 1).

### Changes in metabolic markers

Table 2 shows the changes in the other laboratory outcomes measured at baseline and 52 weeks. After treatment with anagliptin or sitagliptin, high-molecular weight adiponectin levels increased significantly in the anagliptin groups, while there was no significant change in the sitagliptin groups. The FIB-4 index increased significantly in the sitagliptin group, whereas there was no obvious change in the anagliptin group. The absolute changes in these variables did not differ between the two treatment groups. In addition, in the randomly selected patients no significant changes or group differences were observed in lipoprotein (a), RLP-C, and MDA-LDL concentrations.

**Table 2:** Changes in the variables from baseline to 52 weeks

	Anagliptin (*n* = 177)			Sitagliptin (*n* = 176)			*P* value^a^
	Baseline	52 week	*P* value	Baseline	52 week	*P* value	
Low-density lipoprotein cholesterol, mg/dL	112 ± 22	108 ± 22	0.01	109 ± 22	111 ± 22	0.23	0.01
Total cholesterol, mg/dL	191 ± 29	186 ± 27	0.03	186 ± 28	190 ± 28	0.02	0.001
Triglyceride, mg/dL	148 ± 77	155 ± 84	0.42	129 ± 68	136 ± 82	0.19	0.80
Adiponectin, μg/mL	4.4 ± 4.4	4.7 ± 5.2	0.01	4.7 ± 4.9	5.0 ± 5.8	0.14	0.23
Aspartate aminotransferase, IU/L	28 ± 18	27 ± 18	0.60	23 ± 9	26 ± 19	0.02	0.05
Alanine aminotransferase, IU/L	29 ± 19	28 ± 20	0.40	22 ± 13	23 ± 15	0.41	0.24
Platelets, 10^4^/μL	22 ± 6	21 ± 6	0.14	22 ± 5	22 ± 6	0.92	0.23
FIB-4 index	1.8 ± 0.9	1.8 ± 0.9	0.36	1.7 ± 0.8	1.9 ± 1.3	0.03	0.19
Lipoprotein (a), mg/dL^b^	11.2 ± 9.4	11.3 ± 9.1	0.81	14.0 ± 11.6	14.4 ± 12.3	0.39	0.65
Malondialdehyde-modified low-density lipoprotein, U/L^b^	162.9 ± 51.3	153.5 ± 50.9	0.05	152.3 ± 42.4	153.6 ± 45.6	0.80	0.13
Remnant-like particle cholesterol, mmol/L^b^	7.6 ± 4.9	8.8 ± 6.8	0.07	6.6 ± 4.5	7.7 ± 5.4	0.04	0.91

Data are expressed as mean ± standard deviation

^a^For group difference in absolute change from baseline to 52 weeks

^b^Indicates measured in randomly selected cohort (*n *=50 on anagliptin, 50 on sitagliptin)

## Discussion

Recent global clinical guidelines recommend more intensive lipid management in patients with a high cardiovascular risk, such as those with a history of coronary artery disease or T2D [10]. A previous meta-analysis showed clearly that a 1 mmol/L reduction in LDL-C level was associated with a 9% decrease in mortality in patients with diabetes [11]. There is also evidence that treatment with ezetimibe added to a statin is more effective for improving cardiovascular outcomes in patients with acute coronary syndrome and diabetes than those without diabetes [12]. These findings indicate that additive reduction in LDL-C levels with non-statin medications has a large impact on cardiovascular prognosis, especially in patients with diabetes at a high cardiovascular risk.

The class of DPP-4 inhibitors is known to be potentially associated with a beneficial effect on cholesterol levels [13, 14]. Several clinical trials have also shown that anagliptin consistently decreased serum cholesterol levels, including LDL-C, [4, 15, 16]. The LDL-C-lowering effect of anagliptin at 24 weeks was comparable to that of alogliptin, although the anagliptin-mediated reduction in LDL-C level was associated with suppression of apolipoprotein B-100 synthesis in patients with T2D [3]. On the other hand, sitagliptin is also known to decrease serum cholesterol levels in patients with T2D [17–19]. Masuda et al. [18] found that 12 weeks of sitagliptin treatment improved lipid profiles accompanied by reductions in several atherogenic remnant lipoproteins. Kutoh et al. [19] also reported that sitagliptin down-regulated high free fatty acid (FFA) levels and reduced atherogenic cholesterol levels. Furthermore, in experimental T2D model rats sitagliptin ameliorated left ventricular diastolic dysfunction by shifting FFA towards glucose utilization in cardiomyocytes in conjunction with a reduction in lipolysis. In T2D patients without a history of atherosclerotic diseases another DPP-4 inhibitor, vildagliptin, decreased LDL-C, although there was no significant difference in changes in LDL-C between the vildagliptin and metformin groups [20]. Therefore, DPP-4 inhibitors are likely to have a unique effect of reducing LDL-C levels associated with beneficial impacts on lipid profiles. Nevertheless, a meta-analysis of randomized clinical trials showed no significant difference in the changes of LDL-C levels between sitagliptin (alone or in combination) and controls [21]. Furthermore, little is known about intra-class differences in the LDL-C-lowering effect and the mechanisms by which DPP-4 inhibitors influence lipid metabolism in T2D patients even under statin treatment.

To date, no head-to-head clinical study to compare these changes between anagliptin and sitagliptin has been reported, with the strength of the REASON trial being that it was the first study designed specifically to investigate these endpoints between anagliptin and sitagliptin in a clinical setting [2]. Regarding a possible mechanism for this effect, a pilot clinical study in drug-naïve patients with T2D by Aoki et al. [7] showed that anagliptin (without a comparator) decreased serum levels of lathosterol without affecting cholesterol absorption markers, such as campesterol. In the present study where all participants had been receiving background medications for dyslipidemia, the serum level of lathosterol did not change during the 52 weeks of anagliptin treatment, whereas it increased significantly during treatment with sitagliptin. Schonewille et al. [22] have reported previously a statin-induced increase in hepatic cholesterol synthesis in mice, that may partly account for our findings. It is also known that chronic statin administration generally leads to a compensatory increase in intestinal cholesterol absorption [6]. Increases in the serum level of campesterol observed in both treatment groups may therefore indicate that there was no obvious effect of both DPP-4 inhibitors on the cholesterol absorption pathway. Therefore, the changes we observed in markers of lipid metabolisms may be, in part, affected by background statin treatment, with our findings suggesting that anagliptin treatment could at least attenuate excess hepatic cholesterol synthesis compared to that induced by sitagliptin.

A recent experimental study in LDL receptor-deficient mice demonstrated anagliptin down-regulated sterol regulatory element-binding protein-2, a transcriptional factor related to hepatic lipid synthesis [23]. In our study, in addition to attenuating a cholesterol synthesis marker, we found a significant increase in serum adiponectin levels in the anagliptin group, but not in the sitagliptin group. The FIB-4 index, a marker of hepatic fibrosis, also did not change in the anagliptin group, whereas it increased significantly in the sitagliptin group. Lower levels of adipokines, including adiponectin, are known to be associated in the pathophysiology of obesity-related liver diseases [24]. Taken together, these findings suggest that anagliptin exerts a hepato-protective effect beyond its glycemic-lowering action.

Anagliptin is known to have the same glycemic efficacy and safety as sitagliptin as add-on therapy in patients with T2D [25]. A similar result was obtained in the REASON trial even in T2D patients at high cardiovascular risk who were on statin therapy [2]. Therefore, given the additional LDL-C-lowering effect and possible hepato-protective action of anagliptin, the agent may have beneficial effects on lipid metabolism and may have clinical advantages when choosing a glucose-lowering agent in T2D patients who require further treatment to achieve the lipid levels necessary to reduce cardiovascular risk. However, because we observed no significant changes or group differences in other atherogenic lipid parameters, such as lipoprotein (a), RLP-C, and MDA-LDL, in our randomly selected samples, further long-term studies are required to examine whether anagliptin has favorable effects on atherosclerosis and incident cardiovascular events in the observation.

### Limitation

First, because this study was a sub-analysis of the REASON trial it has the same limitations as the original trial [2]. Therefore, the sample size was estimated for the primary and important secondary endpoints of the REASON trial, and the sample size of this sub-analysis might not be sufficiently large to detect clinically meaningful differences in the measurements. Second, no follow-up data on lipid metabolism markers were available during the study period (e.g., 12, 24, and 36 weeks). Our analyses also did not include any data on changes in the full lipoprotein profiles. Third, the statins administered to subjects were not matched between the two groups and this may have influenced our findings. Furthermore, changes in the markers of cholesterol synthesis and absorption are known to vary according to statin dose [26, 27]. However, we have no information on the doses of statins administered to the subjects, although the prevalence of strong statins did not differ between the sitagliptin and anagliptin groups. Fourth, we have no information on the degree of and change in non-pharmacological therapy such as diet and exercise during the study period. However, the effects of imbalance of these factors should be small because this study was originally randomized clinical trial. Finally, because the participants in the REASON trial were all Asian people, it remains uncertain whether or not our findings can be extrapolated to other ethnicities.

## Conclusion

Our findings suggest that anagliptin reduces LDL-C levels by suppressing excess cholesterol synthesis in patients with T2D even in combination with statin therapy. However, further research is needed to assess whether anagliptin specifically affects lipid metabolisms and to examine its profound mechanisms in greater detail.

## Supplementary information

**Additional file 1.** Changes in the lipid metabolism markers in patients who had not received ezetimibe at baseline. Comparison of (A) campesterol, (C) sitosterol, (E) lathosterol at 0 and 52 weeks and absolute change of (B) campesterol, (D) sitosterol, (F) lathosterol at 52 weeks.

## Data Availability

The datasets analyzed during the current study are available from the corresponding author on reasonable request (tanakaa2@cc.saga-u.ac.jp).

## Abbreviations

DPP-4: dipeptidyl peptidase-4
FFA: free fatty acid
LDL-C: low-density lipoprotein cholesterol
MDA-LDL: malondialdehyde-modified low-density lipoprotein
RLP-C: remnant-like particle cholesterol
T2D: type 2 diabetes

## References

[1] Wang Y, Lammi-Keefe CJ, Hou L, Hu G (2013). Impact of low-density lipoprotein cholesterol on cardiovascular outcomes in people with type 2 diabetes: a meta-analysis of prospective cohort studies. Diabetes Res Clin Pract.

[2] Morimoto T, Sakuma I, Sakuma M, Tokushige A, Natsuaki M, Asahi T, Shimabukuro M, Nomiyama T, Arasaki O, Node K (2019). Randomized evaluation of anagliptin vs sitagliptin on low-density lipoproteiN cholesterol in diabetes (REASON) trial: a 52-week, open-label, randomized clinical trial. Sci Rep.

[3] Kurozumi A, Okada Y, Arao T, Kobayashi T, Masuda D, Yamashita S, Tanaka Y (2018). Comparison of effects of anagliptin and alogliptin on serum lipid profile in type 2 diabetes mellitus patients. J Diabetes Investig.

[4] Kakuda H, Kobayashi J, Kakuda M, Yamakawa J, Takekoshi N (2015). The effect of anagliptin treatment on glucose metabolism and lipid metabolism, and oxidative stress in fasting and postprandial states using a test meal in Japanese men with type 2 diabetes. Endocrine.

[5] Reihner E, Rudling M, Stahlberg D, Berglund L, Ewerth S, Bjorkhem I, Einarsson K, Angelin B (1990). Influence of pravastatin, a specific inhibitor of HMG-CoA reductase, on hepatic metabolism of cholesterol. N Engl J Med.

[6] van Himbergen TM, Matthan NR, Resteghini NA, Otokozawa S, Ai M, Stein EA, Jones PH, Schaefer EJ (2009). Comparison of the effects of maximal dose atorvastatin and rosuvastatin therapy on cholesterol synthesis and absorption markers. J Lipid Res.

[7] Aoki K, Ijima T, Kamiyama H, Kamiko K, Terauchi Y (2015). Anagliptin decreases serum lathosterol level in patients with type 2 diabetes: a pilot study. Expert Opin Pharmacother.

[8] Ueda S, Shimabukuro M, Arasaki O, Node K, Nomiyama T, Morimoto T (2018). Effect of anagliptin and sitagliptin on low-density lipoprotein cholesterol in Type 2 diabetic patients with dyslipidemia and cardiovascular risk: rationale and study design of the REASON trial. Cardiovasc Drugs Ther.

[9] Kim BK, Kim DY, Park JY, Ahn SH, Chon CY, Kim JK, Paik YH, Lee KS, Park YN, Han KH (2010). Validation of FIB-4 and comparison with other simple noninvasive indices for predicting liver fibrosis and cirrhosis in hepatitis B virus-infected patients. Liver Int.

[10] Grundy SM, Stone NJ, Bailey AL, Beam C, Birtcher KK, Blumenthal RS, Braun LT, de Ferranti S, Faiella-Tommasino J, Forman DE (2019). 2018 AHA/ACC/AACVPR/AAPA/ABC/ACPM/ADA/AGS/APhA/ASPC/NLA/PCNA guideline on the management of blood cholesterol: executive summary: a report of the American College of Cardiology/American Heart Association Task Force on Clinical Practice Guidelines. Circulation.

[11] Kearney PM, Blackwell L, Collins R, Keech A, Simes J, Peto R, Armitage J, Baigent C (2008). Efficacy of cholesterol-lowering therapy in 18,686 people with diabetes in 14 randomised trials of statins: a meta-analysis. Lancet (London, England).

[12] Giugliano RP, Cannon CP, Blazing MA, Nicolau JC, Corbalan R, Spinar J, Park JG, White JA, Bohula EA, Braunwald E (2018). Benefit of adding ezetimibe to statin therapy on cardiovascular outcomes and safety in patients with versus without diabetes mellitus: results from IMPROVE-IT (Improved Reduction of Outcomes: Vytorin Efficacy International Trial). Circulation.

[13] Monami M, Lamanna C, Desideri CM, Mannucci E (2012). DPP-4 inhibitors and lipids: systematic review and meta-analysis. Adv Ther.

[14] Ahn CH, Kim EK, Min SH, Oh TJ, Cho YM (2017). Effects of gemigliptin, a dipeptidyl peptidase-4 inhibitor, on lipid metabolism and endotoxemia after a high-fat meal in patients with type 2 diabetes. Diabetes Obes Metab.

[15] Nishio S, Abe M, Ito H (2015). Anagliptin in the treatment of type 2 diabetes: safety, efficacy, and patient acceptability. Diabetes Metab Syndr Obes.

[16] Chiba Y, Yamakawa T, Tsuchiya H, Oba M, Suzuki D, Danno H, Takatsuka Y, Shigematsu H, Kaneshiro M, Terauchi Y (2018). Effect of anagliptin on glycemic and lipid profile in patients with type 2 diabetes mellitus. J Clin Med Res.

[17] Shigematsu E, Yamakawa T, Kadonosono K, Terauchi Y (2014). Effect of sitagliptin on lipid profile in patients with type 2 diabetes mellitus. J Clin Med Res.

[18] Masuda D, Kobayashi T, Sairyou M, Hanada H, Ohama T, Koseki M, Nishida M, Maeda N, Kihara S, Minami T (2018). Effects of a dipeptidyl peptidase 4 inhibitor sitagliptin on glycemic control and lipoprotein metabolism in patients with type 2 diabetes mellitus (GLORIA Trial). J Atheroscler Thromb.

[19] Kutoh E, Wada A, Hayashi J (2018). Regulation of free fatty acid by sitagliptin monotherapy in drug-naive subjects with type 2 diabetes. Endocr Pract.

[20] Kitao N, Miyoshi H, Furumoto T, Ono K, Nomoto H, Miya A, Yamamoto C, Inoue A, Tsuchida K, Manda N (2017). The effects of vildagliptin compared with metformin on vascular endothelial function and metabolic parameters: a randomized, controlled trial (Sapporo Athero-Incretin Study 3). Cardiovasc Diabetol.

[21] Fan M, Li Y, Zhang S (2016). Effects of sitagliptin on lipid profiles in patients with type 2 diabetes mellitus: a meta-analysis of randomized clinical trials. Medicine.

[22] Schonewille M, de Boer JF, Mele L, Wolters H, Bloks VW, Wolters JC, Kuivenhoven JA, Tietge UJ, Brufau G, Groen AK (2016). Statins increase hepatic cholesterol synthesis and stimulate fecal cholesterol elimination in mice. J Lipid Res.

[23] Yano W, Inoue N, Ito S, Itou T, Yasumura M, Yoshinaka Y, Hagita S, Goto M, Nakagawa T, Inoue K (2017). Mechanism of lipid-lowering action of the dipeptidyl peptidase-4 inhibitor, anagliptin, in low-density lipoprotein receptor-deficient mice. J Diabetes Investig.

[24] Polyzos SA, Kountouras J, Mantzoros CS (2016). Adipokines in nonalcoholic fatty liver disease. Metab Clin Exp.

[25] Jin SM, Park SW, Yoon KH, Min KW, Song KH, Park KS, Park JY, Park IB, Chung CH, Baik SH (2015). Anagliptin and sitagliptin as add-ons to metformin for patients with type 2 diabetes: a 24-week, multicentre, randomized, double-blind, active-controlled, phase III clinical trial with a 28-week extension. Diabetes Obes Metab.

[26] Lamon-Fava S, Diffenderfer MR, Barrett PH, Buchsbaum A, Matthan NR, Lichtenstein AH, Dolnikowski GG, Horvath K, Asztalos BF, Zago V (2007). Effects of different doses of atorvastatin on human apolipoprotein B-100, B-48, and A-I metabolism. J Lipid Res.

[27] Ooi EM, Barrett PH, Chan DC, Nestel PJ, Watts GF (2008). Dose-dependent effect of rosuvastatin on apolipoprotein B-100 kinetics in the metabolic syndrome. Atherosclerosis.

