# Supplementary material for: Differences in lipid metabolism between anagliptin and sitagliptin in patients with type 2 diabetes on statin therapy: a secondary analysis of the REASON trial
Source: Cardiovasc Diabetol. 2019 Nov 16;18:158. doi: 10.1186/s12933-019-0965-3 (PMC6858725; doi:10.1186/s12933-019-0965-3)
Supplement: Supplementary file 1 — Additional file 1. Changes in the lipid metabolism markers in patients who had not received ezetimibe at baseline. Comparison of (A) campesterol, (C) sitosterol, (E) lathosterol at 0 and 52 weeks and absolute change of (B) campesterol, (D) sitosterol, (F) lathosterol at 52 weeks. [file 12933_2019_965_MOESM1_ESM.pptx]

## Slide 1
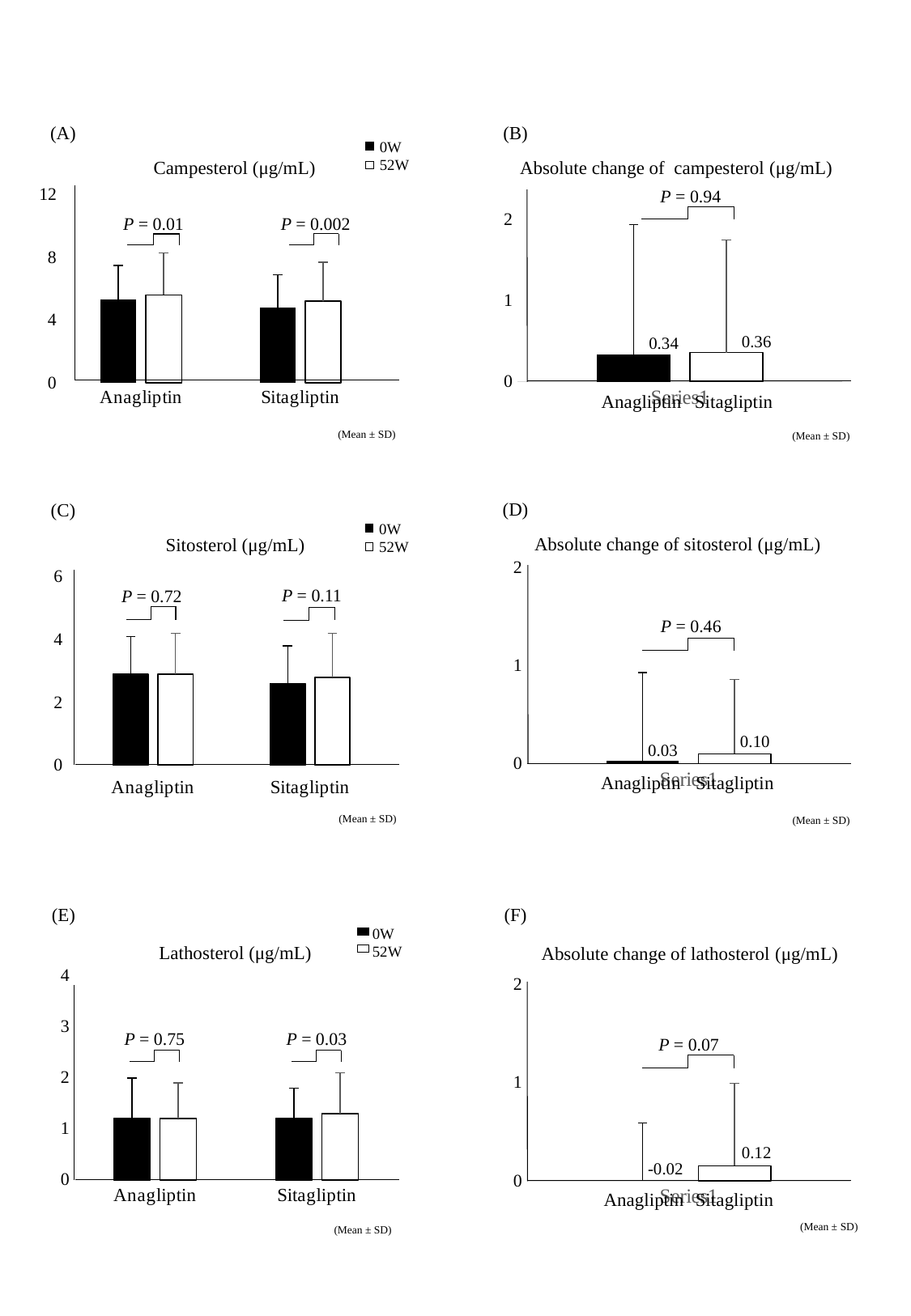

(B)
(A)
0W
52W
Absolute change of campesterol (μg/mL)
Campesterol (μg/mL)
### Chart
| Category | 0W | 52W |
|---|---|---|
| Anagliptin | 5.3 | 5.6 |
| Sitagliptin | 4.8 | 5.2 |P = 0.94
### Chart
| Category | Anagliptin | Sitagliptin |
|---|---|---|
| | 0.34 | 0.36 |P = 0.01
P = 0.002
0.36
0.34
Anagliptin
Sitagliptin
(Mean ± SD)
(Mean ± SD)
(D)
(C)
0W
52W
Absolute change of sitosterol (μg/mL)
Sitosterol (μg/mL)
### Chart
| Category | Anagliptin | Sitagliptin |
|---|---|---|
| | 0.03 | 0.1 |
### Chart
| Category | 0W | 52W |
|---|---|---|
| Anagliptin | 2.9 | 2.9 |
| Sitagliptin | 2.6 | 2.8 |P = 0.11
P = 0.72
P = 0.46
0.10
0.03
Anagliptin
Sitagliptin
(Mean ± SD)
(Mean ± SD)
(E)
(F)
0W
52W
Lathosterol (μg/mL)
Absolute change of lathosterol (μg/mL)
### Chart
| Category | 0W | 52W |
|---|---|---|
| Anagliptin | 1.2 | 1.2 |
| Sitagliptin | 1.2 | 1.3 |
### Chart
| Category | Anagliptin | Sitagliptin |
|---|---|---|
| | 0.01 | 0.152 |P = 0.75
P = 0.03
P = 0.07
0.12
-0.02
Anagliptin
Sitagliptin
(Mean ± SD)
(Mean ± SD)
